# The Rare Large Common Peroneal Nerve's Schwannoma—A Case Report and Literature Review

**DOI:** 10.1155/2024/9397436

**Published:** 2024-05-02

**Authors:** Rudiansyah Harahap, Nurmaliannysa Dwinandia Harahap

**Affiliations:** ^1^Faculty of Medicine, Muhammadiyah University, Semarang, Central Java, Indonesia; ^2^Faculty of Medicine, Sultan Agung Islamic University, Semarang, Central Java, Indonesia

**Keywords:** common peroneal nerve, neurological deficit, schwannoma

## Abstract

Schwannoma in the popliteal fossa is still rare, often diagnosed late because it grows slowly and has no symptoms. It is often misdiagnosed with connective tissue tumors or with neurological disorders originating in the spine or disorders of the peroneal nerve. Schwannoma within the common peroneal nerve is still rare in the popliteal fossa, with most tumor sizes around 2 cm in diameter due to their smaller size of nerve but can cause neurologic disturbance, especially when it is large. And over a long time, it can cause serious complaints like neurological deficits and make surgery difficult by leaving greater sequelae. There is no data yet showing the incidence of schwannoma in the common peroneal nerve. In this case, a 36-year-old woman, for 5 years, feels soreness in the popliteal fossa and pain in the right instep, suspected that a nerve was pinched, due to an abnormality in the spine. As time went on, there was a lump in the fold of the right knee, suspected to be a Baker's cyst. As time went by, the complaint was burning pain in the right instep to the lateral ankle and distal right lower leg, disturbing sleep. Tinel's sign was positive. The right instep has hypoesthesia and a slight drop in the foot. On radiological examination of the right knee, a circumferential mass appeared, measuring 5 cm × 4 cm. The diagnosis is suspicious for a common peroneal nerve tumor. The encapsulated operation to remove the tumor was carried out with a size measuring 5 cm × 4.5 cm × 4 cm. The histopathological examination showed schwannoma. After surgery, the pain disappeared, hypoesthesia and a slight drop in the foot underwent physiotherapy, and stimulation with the result gradually improved. A thorough early examination includes correct and systematic anamnesis, physical examination, and neurological evaluation such as paraesthesia, hypoesthesia, and Tinel's sign; also, additional examinations, such as radiographic, ultrasound, and MRI, are needed for early detection of schwannoma so that delays in diagnosis and surgery can be avoided to prevent neurological deficits.

## 1. Introduction

Connective tissue tumors in the popliteal fossa that often occur are ganglion, lipoma, Baker's cyst, neurofibroma, and schwannoma. Schwannomas are benign tumors, slow growing, solitary, circumferential, encapsulated, arising from Schwann's cell of peripheral nerve, and rarely turn malignant [1, 2]. The diagnosis of schwannoma is often late or misdiagnosed with other tumors, especially with Baker's cyst [3]. The large schwannoma is often presented in the spine [4, 5], head, and neck (25%–45%) [6], while they represent approximately 5% of soft tissue neoplasm [1]. They rarely occur in the lower extremities, and in the popliteal fossa even more rarely [6, 7]. They often affect the posterior tibial nerve at around 8.9% [2, 6], less in the sciatic nerve at around 1% [2, 8], and even less frequently in the common peroneal nerve. The common peroneal nerve due to its smaller size than the posterior tibial nerve and sciatic nerve is even less frequently affected by such tumors, with most schwannomas in this location measuring around 2 cm in diameter [9, 10]. With a size of around 2 cm, it can cause dysfunction in the feet [11]. However, schwannoma in the common peroneal nerve can be very large in this nerve because it was diagnosed very late for 20 years, up to 7 cm, and the potential for significant functional impairment underscores the importance of prompt diagnosis [12].

At first, there were no complaints, then there was a feeling of soreness in the popliteal fossa. If it gets longer, especially if the tumor is large, the compression effect of the schwannoma on the common peroneal nerve gets bigger and causes pain for a long time. It feels hot, like burning in the right instep to the lateral ankle and lower leg. If the area is hit, the pain spreads from the lateral lower leg to the right instep (positive Tinel's sign), and the longer it feels thick (hypoesthesia) [2, 6, 7]. Surgery to remove the tumor is needed [6, 7]. The risk of nerve injury depends on the size and the duration of tumor detection [7, 8]. Early detection is critical not only to mitigate the risk of nerve injury and functional disability but also to facilitate surgical removal of the tumor with optimal outcome. This case report illustrates a rare instance of a large schwannoma within the common peroneal nerve with an emphasis on the challenge associated with its late presentation and the subsequent management strategies employed to address the resulting neurological deficits.

## 2. Case

**Figure 1:**
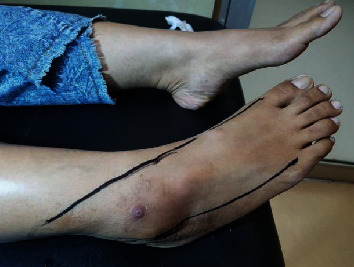
Clinical location complaints of hypoesthesia and burning sensation.

**Figure 2:**
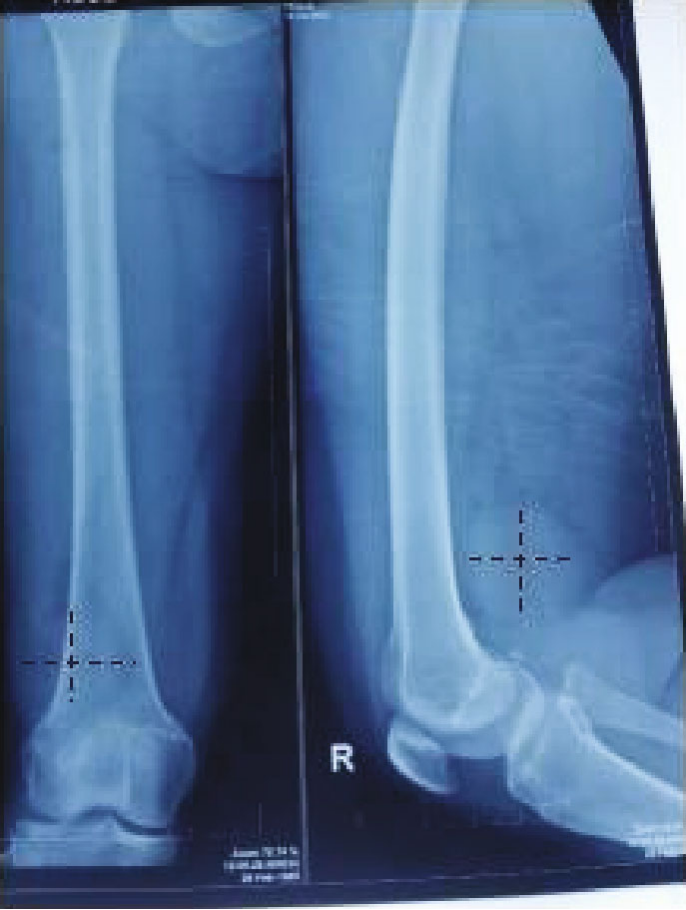
X-ray of the right knee with a mass in the popliteal fossa more laterally, measuring 5 cm × 4 cm.

A 36-year-old woman initially went to a neurologist with complaints of frequent soreness in her right leg, and pain in the right instep, suspected to be a pinched nerve from the spine. Then, the longer the feeling of a lump in the right popliteal fossa, the diagnosis was “Baker's cyst.” Since 5 years ago, the right knee has felt sore, and since 4 years ago, it felt like a lump was getting bigger. The right instep to the lateral side of the ankle and the 1/3 distal of the lower leg sometimes felt hot, like burning, or painful like pulling. Since 2 years ago, it sometimes feels like an electric shock from the fold of the right knee to the right instep along the lateral side of the lower leg if the lump in the right popliteal fossa is hit (Tinel's sign is positive). The lump gets bigger, hurts more often, and interferes with rest or sleep at night. A physical examination showed that the patient was in pain, and no abnormalities were found in the vertebrae or elsewhere. In the right popliteal fossa, a mass was palpable, deep, more lateral, spongy, flat, mobile, single, and tender, and Tinel's sign was positive. Hypoesthesia sensibility in the right instep to the lateral ankle and distal to the right lower leg, and a slight drop in the foot (Figure 1). On radiographic examination of the right knee, a circumscript, single, more lateral mass was seen, measuring approximately 4 cm × 5 cm in the popliteal fossa (Figure 2).

**Figure 3:**
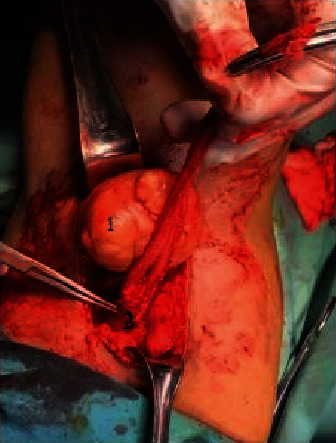
Intraoperative. (1) Round, flat, solitary tumor, attached to the common peroneal nerve. (2) Right common peroneal nerve.

**Figure 4:**
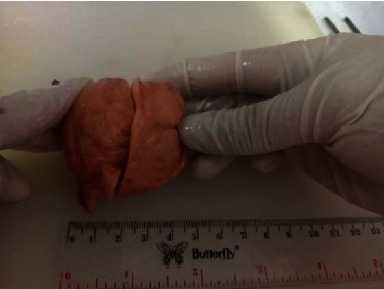
Tumor size was measured, 5 cm × 4.5 cm × 4 cm.

**Figure 5:**
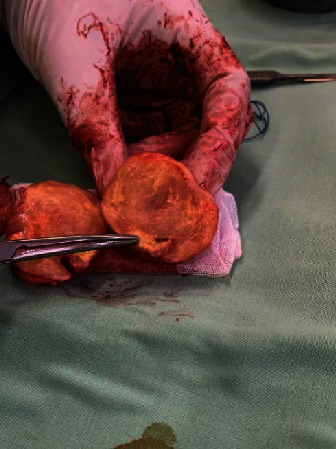
The tumor was sectioned, solid, and brownish, and no necrotic tissue was visible.

**Figure 6:**
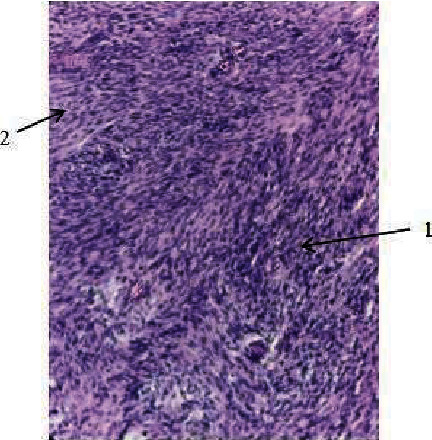
Histopathology results. (1) Palisade elements of the Antoni A cell. (2) The sparse area of cells is called the Antoni B area.

The patient was diagnosed with a right popliteal tumor, suspicious of a peroneus common peripheral nerve tumor. Surgery was performed to remove the tumor, prone position, and lazy S incision. The tumor appeared solitary, spongy, originating or attached to the right common peroneal nerve, flat, and mobile. We opened the nerve sheath capsule in the same direction as the nerve (fascicle) without cutting the nerve pathway/fascicle, removing the tumor (Figure 3). The tumor was measured with results of 5 cm × 4.5 cm × 4 cm (Figure 4). The tumor sectioned was solid, flat, and almost homogeneous, with no visible tissue necrosis and brownish-yellow color (Figure 5). A histopathological examination was carried out with the result of “cellular schwannoma” (Figure 6). Postoperatively, neurological deficits in the form of hypoesthesia and drop foot were discovered, and pain and burning disappeared. Physiotherapy and stimulation of the right common peroneal nerve gradually reduced hypoesthesia and foot drop, but need further evaluation.

## 3. Discussion

Benign peripheral nerve sheath tumors are classified into schwannoma and neurofibroma [1]. Schwannoma is the most common peripheral nerve sheath tumor. Other names for schwannoma are neurilemoma, neurocytoma, peripheral glioma, perineural fibroblastoma, neurinoma, and neurolemmoma [2]. It is most often called neurilemoma [2]. It is often misdiagnosed with other tumors such as Baker's cyst, ganglion, lipoma, fibroma, or xanthoma because it is often asymptomatic, grows slowly, soft, and painless [13]. It is most often misdiagnosed with Baker's Cyst because it is a soft tissue tumor in the popliteal which is the most common [14]. The accuracy of diagnosing schwannoma in the superior extremities is around 31% [15]. White reported that the accuracy of diagnosing schwannoma was five out of thirty-two cases [14]. Kehoe, Reid, and Semple reported that the accuracy of preoperative diagnosis of schwannoma based on anatomical pathology results was 1:88 [16]. The literature suggests that schwannomas are most frequently present in individuals aged 20–50 with no distinct gender predilection [1, 8]. Although these tumors are more common in the head, neck [17, 18], vertebrae, and upper extremities [19], their occurrence in the lower extremities is less common, accounting for 25% of all benign tumors in this region [20]. Knight, Birch, and Pringle stated that 8.9% of benign solitary schwannoma occur in the tibial nerve [21]. In the sciatic nerve, it is 1% [22–26]. Specifically, schwannomas of the common peroneal nerve are rare with most documented cases measuring between 1 and 4 cm in diameter (11–13 cases/84.6%) [11, 27–31]. Meanwhile, two of the thirteen cases are large (15.38%) (Table 1) [12, 32].

In this case, the schwannoma in the peroneus communis nerve was large in size, 5 cm × 4.5cm × 4 cm, and in a long time (5 years), causing quite serious symptoms in the form of burning pain in the instep to the lateral ankle and distal right lower leg, which disturbed sleep, as well as neurological deficits in the form of hypoesthesia. This is likely caused by large tumor compression on the peroneus communis nerve [33], which is relatively smaller than the tibialis posterior nerve and sciatic nerve, but sometimes there is schwannoma that continues or fuses with the fascicle which causes a high risk of nerve damage during excision or surgery [27]. Kim et al. found that two out of thirty operated patients experienced persistent motor complaints and sensory disturbances [34]. From Table 1, evaluating fourteen cases of schwannoma in the common peroneal nerve from 2014 to 2022, it was found that eight cases (57.1%) had neurological deficits, four cases had motor deficits, and four cases had sensory deficits. Tumors that have been there for a long time and large in the relatively small peroneal nerve, with nerve disorders in the form of burning pain, radiating pain, and hypoesthesia, make surgery difficult and worsen postoperative results in the form of neurological deficits such as hypoesthesia and slight drop foot. The burning pain disappeared, and the patient could sleep soundly. Postsurgery continued with physiotherapy and stimulation right peroneus communis nerve with the result that the neurological deficit gradually decreased and further evaluation was carried out.

Based on the literature and cases above, schwannoma in the popliteal, especially in the common peroneal nerve, is often diagnosed late because it is misdiagnosed with other soft tissue tumors, causing severe symptoms until neurological deficits arise and large tumors complicate surgery with poor results or persistent neurological deficits. To prevent late diagnosis of schwannoma in the popliteal, especially in the peroneus communis nerve, a systematic and correct history and physical examination of the general and local status, including neurological statuses such as soreness or lump and Tinel's sign in the popliteal fossa, hyperesthesia, paraesthesia, and burning sensation, hypoesthesia on the back of the sole and lateral ankle to the lower leg, supporting examinations such as radiography, ultrasonography, and MRI, as well as preparation for surgical planning to facilitate surgery and prevent neurological deficits.

## 4. Conclusion

Schwannoma of the common peroneal nerve in the popliteal fossa is relatively rare and is often misdiagnosed with Baker's cyst at first because it grows slowly, is solitary, and has no symptoms, so it is diagnosed too late. Delay or wrong diagnosis causes clinical symptoms to become more severe and tumors to become larger, resulting in increasingly severe neurological disorders and deficits. In this case, early diagnosis is needed, it can be made with a thorough anamnesis or patient's history and physical examination including evaluation of peripheral nerves such as hyperesthesia, paraesthesia, burning sensation, hypoesthesia, and “Tinel's sign.” Additional examinations such as radiography, ultrasound, and MRI are very supportive so that treatment is faster and more accurate for peripheral nerve tumors in the hope of reducing the risk of neurological deficits. Encapsulated surgery was carried out to evacuate the tumor without dissection of the nerve, only opening the perineurum or capsule, a definite diagnosis by histopathological examination. Physiotherapy and stimulation of the common peroneal nerve were carried out after surgery to improve the neurological deficit.

## Figures and Tables

**Table 1:** Case of schwannoma in the common peroneal nerve.

**No.**	**Writer/researcher**	**Year**	**Patient age**	**Length of diagnosis**	**Tumor size**	**Initial complaint**	**Tinel's sign**	**Neurological deficits**	**Therapy**	**Publication**
1	Cho et al. [32]	2014	—	No data	6.2 × 3.8 × 2.8 cm	Drop foot	No data	Drop foot	Surgery	*Annals of Clinical Neurophysiology*, volume 16 (2); 2014
2	Öz et al. [9]	2017	39 years old	3 months	3 × 2 cm	Knee pain	+	—	Surgery	*Research Gate Orthopedic Review*, 2017; volume 9:6825
3	Tan et al. [27]	2017	50 years old	6 months	3 × 2 cm	Pain and swelling in the right popliteal fossa	+	Weak ankle dorsiflexion	Surgery	*Journal of Surgical Academia*, 2017, 7(2), PP 24-27
4	Milenković and Mitković [28]	2018	41 years old	5 months	3 × 2.5 cm	Knee pain	No data	—	Surgery	*Hippokratia*, 2018 Apr-June; 22(2):91. PMCID: PMC 6548524/PMID: 31217682
5	Budohoski et al. [10]	2018	62 years old	2 years	No data	A palpable mass in the lateral popliteal fossa	No data	—	Surgery	*NIH PubMed*, 2018. PMID: 28521057. DOI:10.1093/ounce/Opxo66
6	Vetrano et al. [11]	2020	23 years old	1 year	1.4 × 1.6 cm	Pain and left popliteal nodule	No data	Weakness in the left foot extension	Surgery	*Surgical Neurology International*, 2020. 11C413J
7	Panchariya et al. [29]	2020	12 years old	5 years	3 × 3 cm	Swelling in the right popliteal fossa	No data	—	Surgery	*IP Indian Journal of Neuroscience*, 2020; 6(4): 330-332
8	Shariq, Radha, and Konan [30]	2021	60 years old	2 years	3 × 4 cm	Intermittent left knee pain	No data	—	Surgery	*BMJ Case Reports* Volume 2012
9	Georgiev, Anoniev, and Slavchev [12]	2021	81 years old	20 years	7 cm	Slow-growing mass at posterolateral popliteal	No data	—	Surgery	*Current Problems in Cancer: Case Reports*, volume 3, March 2021, 100061
10	Andreani et al. [31]	2022 (2016–2020)	39 years old	11 months	2 cm	Paraesthesia	+	Sensory deficit	Surgery	*J. Musculoskelet Neuronal Interest*, 2022, 22(1):87-92. PMCID: PMC8919662/PMID: 35234163
			72 years old	3 months	3 cm	Tumefaction (tumor)	±	Sensory deficit	Surgery	
			54 years old	12 months	4.2 cm	Paraesthesia	+	Sensory deficit	Surgery	
			48 years old	5 months	2.5 cm	Paraesthesia	+	Sensory deficit	Surgery	
			44 years old	9 months	3.9 cm	Weakness	+	Sensory deficit, weakness	Surgery	
